# A Phase 2 Clinical Trial of Combination Nivolumab, Ipilimumab, and Paclitaxel in Patients With Untreated Metastatic NSCLC: The OPTIMAL Trial

**DOI:** 10.1016/j.jtocrr.2022.100337

**Published:** 2022-05-17

**Authors:** Jeffrey M. Clarke, Lin Gu, Xiaofei F. Wang, Thomas E. Stinchcombe, Marvaretta M. Stevenson, Sundhar Ramalingam, Afreen Shariff, Jennifer Garst, Andrew B. Nixon, Scott J. Antonia, Jeffrey Crawford, Neal E. Ready

**Affiliations:** a Duke Cancer Institute, Durham, North Carolina; b Department of Medicine, Duke University School of Medicine, Durham, North Carolina; c Department of Biostatistics and Bioinformatics, Duke University School of Medicine, Durham, North Carolina; d Duke Raleigh Hospital, Duke Cancer Institute, Durham, North Carolina

**Keywords:** Non–small cell lung cancer, Immunotherapy, Nivolumab, Ipilimumab, Chemotherapy, Clinical trial

## Abstract

**Introduction:**

Most patients with advanced NSCLC will experience disease progression and death within 2 years. Novel approaches are needed to improve outcomes.

**Methods:**

We conducted an open-label, nonrandomized, phase 2 trial in patients with treatment-naive, advanced NSCLC to assess the safety and efficacy of nivolumab 360 mg every 3 weeks, ipilimumab 1 mg/kg every 6 weeks, and four to six cycles of paclitaxel 80 mg/m^2^ on days 1 and 8 of every 21-day treatment. The primary end point of the study was median progression-free survival (PFS), with secondary end points of safety, objective response rate, and median overall survival (OS).

**Results:**

A total of 46 patients underwent consent and received treatment. The median age was 66 (range: 48–82) years, most had adenocarcinoma (63%), and 50% (23) had programmed death-ligand 1 greater than or equal to 1%. The median follow-up on the study as of October 2021 was 19 months. The primary end point of median PFS was 9.4 months (95% confidence interval [CI]: 5.9–16.6) in all patients regardless of programmed death-ligand 1 expression. The objective response rate for patients in the study was 47.8% (95% CI: 33.4–62.3). The 12-month OS rate was 69.5% (95% CI: 53%–81%), and median OS was not yet reached. Treatment-related grade greater than or equal to 3 adverse events was found in 54.3% of the patients.

**Conclusions:**

The toxicity observed was consistent with other reported chemo-immunotherapeutic combinations and was manageable. The primary end point of exceeding median PFS of 9 months was achieved with nivolumab, ipilimumab, and weekly paclitaxel and should be evaluated further in a randomized trial.

## Introduction

The incorporation of immune checkpoint-based therapies targeting the programmed cell death protein 1 (PD-1) or programmed death-ligand 1 (PD-L1) axis earlier into the treatment paradigm of advanced NSCLC has resulted in considerable long-term survival benefit for patients. Approved strategies generally leverage immune checkpoint blockade of PD-1 or PD-L1 as a monotherapy, part of dual blockade with anti–CTLA-4 treatment, or as combination with chemotherapy. Although several agents were found to have frontline efficacy as monotherapy and remain well tolerated, the clinical benefit has largely been dependent on PD-L1 tumor expression, and early disease progression will occur in a large subset of patients.^1^, ^2^, ^3^ Combination regimens using platinum-doublet chemotherapy with anti–PD-1 or PD-L1 therapy were found to have robust response rates and improvement in survival outcomes across subgroups by PD-L1 expression.^4^, ^5^, ^6^ The addition of chemotherapy to immunotherapy has well-described advantageous immunologic effects in the tumor microenvironment including provoking immunogenic cell death, activating dendritic cells, facilitating cross-priming of T cells, and reducing regulatory T cells and myeloid-derived suppressor cells.^7^^,^^8^ Nevertheless, platinum-based doublet chemotherapy and immune checkpoint treatment are often used at the expense of increased toxicity compared with immunotherapy alone.

Dual checkpoint inhibition with nivolumab and ipilimumab has been evaluated in multiple clinical trials, and most extensively for NSCLC in the large, phase 3 CheckMate-227 study.^9^ The addition of anti–CTLA-4 blockade to nivolumab resulted in durable improvement in survival relative to chemotherapy in both PD-L1 expressed and nonexpressed groups. The median progression-free survival (PFS) for both PD-L1 greater than or equal to 1% and less than 1% groups was 5.1 months with median overall survival (OS) of approximately 17.1 months. Most strikingly, approximately a third of patients had durable response to treatment at 4 years of follow-up with median duration of response of more than 23 months for patients with tumors having PD-L1 greater than or equal to 1% expression.^10^ On the basis of the results of this trial, combination nivolumab and ipilimumab is now a Food and Drug Administration–approved regimen for EGFR/ALK alteration-negative NSCLC with PD-L1 greater than or equal to 1% in the United States. The CheckMate-9LA study used a chemotherapy backbone with a platinum doublet combined with nivolumab/ipilimumab, where chemotherapy was given for two cycles at initiation of treatment. The quadruple drug regimen was found to have significant improvement in PFS, OS, with an overall acceptable toxicity profile.^11^ Importantly, survival benefit was found across all PD-L1 subgroups, leading to its approval as a standard treatment option in the frontline setting.

Building on the CheckMate-227 and CheckMate-9LA trials, we designed a single-arm phase 2 study of dual immune checkpoint blockade with nivolumab and ipilimumab in combination with weekly paclitaxel for advanced, untreated NSCLC. The principal rationale of adding low-dose weekly chemotherapy was to prevent early disease progression, as found with other immunotherapy-only approaches, and facilitate immunogenic tumor cell death. In addition, the approach of using a weekly dosed taxane would minimize significant platinum chemotherapy-related toxicity. The hypothesis of the study was to improve PFS as compared with standard combination nivolumab and ipilimumab and result in a median PFS (mPFS) of 9 months or more. The duration of chemotherapy exposure in the study was limited to 4 to 6 cycles to prevent accumulation of further toxicity, including fatigue, neuropathy, and cytopenias. Finally, an added advantage of this approach was to preserve platinum-based chemotherapy as a second-line treatment option in the event of progression on frontline therapy.

## Materials and Methods

### Study Design

The OPTIMAL (Thoracic Oncology Program 1705) study was conducted as an open-label, single-arm, phase 2 trial designed to assess the safety and efficacy of nivolumab and ipilimumab in combination with weekly paclitaxel on days 1 and 8 every 21 days in patients with treatment-naive advanced NSCLC. Informed consent was obtained from patients with metastatic or recurrent and not curable NSCLC. Patients were enrolled after complete and extensive medical history, baseline physical examination, and clinical assessment to ensure subject eligibility requirements. The dosing regimen was nivolumab 360 mg every 3 weeks, ipilimumab 1 mg/kg every 6 weeks, and paclitaxel 80 mg/m^2^ on days 1 and 8 of every 21-day treatment cycle. Paclitaxel was stopped after a total of four to six cycles of treatment, based on decision of treating provider or unacceptable toxicity. Patients could continue to receive nivolumab and ipilimumab until they experience unacceptable treatment-related toxicity, disease progression, or once they have been treated for 2 years.

### Patients

Patients (aged ≥18 y) were eligible for enrollment with histologically confirmed stage IV or recurrent NSCLC squamous or nonsquamous histology (seventh edition), with no prior systemic anticancer therapy given as primary therapy for advanced or metastatic disease. Prior adjuvant chemotherapy, neoadjuvant chemotherapy, or chemoradiotherapy was permitted as long as the last administration of the prior regimen occurred at least 6 months before study enrollment. Patients with known EGFR, ALK, or ROS1 alterations must have received at least one prior targeted agent. Patients were eligible for enrolment regardless of PD-L1 testing status or results. Performance status was required to be Eastern Cooperative Oncology Group 0 to 1 with adequate end organ and marrow function. Patients were excluded if requiring radiation within 14 days of treatment, having prior intolerance of PD-1 or PD-L1 axis drug, or having known autoimmune conditions requiring systemic immune suppression therapy other than prednisone < 10 mg daily. Untreated brain metastases were allowed if subject did not require corticosteroids or anticonvulsant therapy. Concurrent severe or uncontrolled medical conditions were excluded including active infection requiring antibiotics, recent myocardial infarction, stroke, or clinically significant heart disease. Patients with known history of the participating center approved the study. Human immunodeficiency virus seropositivity or known acquired immunodeficiency syndrome, active hepatitis C virus, and acute or chronic active hepatitis B infection were excluded. The institutional review board of the participating center approved the study, and this trial was conducted in accordance with Good Clinical Practice guidelines and the provisions of the Declaration of Helsinki. Patients were required to provide informed consent before any study-related procedures. This trial was registered at ClinicalTrials.gov (NCT03573947).

### Procedures

Efficacy was assessed by radiographic imaging (computed tomography and/or magnetic resonance imaging) every 6 weeks (±7 d) up to week 48 and then every 12 weeks until documented disease progression using Response Evaluation Criteria in Solid Tumors version 1.1. Safety assessments were performed every 3 weeks, as clinically indicated, 35 days after the last dose of the study drug, and 100 days after the last dose of the study drug. These assessments include vital signs, Eastern Cooperative Oncology Group performance status, medical history, physical examination, complete blood cell count, biochemistry, creatinine, aspartate transaminase, alanine transaminase, and bilirubin. Thyroid-stimulating hormone testing will be performed at regular intervals. Adverse events were recorded using National Cancer Institute Common Terminology Criteria for Adverse Events version 4.03. General symptom management and supportive care as clinically indicated to ensure optimal patient care were provided.

### Outcomes

The primary objective of this trial was to estimate the PFS for the combination nivolumab, ipilimumab, and paclitaxel in untreated, metastatic NSCLC. The PFS was measured from the date of registration to the date of progression or death from any cause, whichever comes first. Secondary objectives included estimating the objective response rate (ORR) and OS with the study combination. In addition, the safety and adverse event profile was described for combination of nivolumab, ipilimumab, and paclitaxel in untreated, metastatic NSCLC. Baseline tumor and sequential peripheral blood specimens were collected for future exploratory analysis to explore correlation between baseline and treatment-related changes in immune correlates and clinical outcome.

### Statistical Analysis Plan and Methods

The primary objective of the study was to determine whether the combination regimens will improve PFS relative to historical control from the CheckMate-227 study. The hypothesis tested was that treatment with the combination of nivolumab (Opdivo), ipilimumab (Yervoy), and paclitaxel (Taxol) would be associated with mPFS of 9 months or more. If mPFS is less than 6 months, then we conclude that the combination regimen is not worthy of further investigation (H0: mPFS ≤ 6 mo versus H1: mPFS ≥ 9 mo). Using one-sided, one-sample log-rank test at a significant level of 0.1, the study with 46 subjects has approximately 85% power to test the alternative hypothesis. Taking 5% of ineligibility or dropout into consideration, up to 49 patients were allowed to be enrolled in the study.

The Kaplan-Meier product limit estimator was used to graphically characterize PFS. From these product limit estimates, the mPFS and the rate of PFS at 12 months and their corresponding confidence intervals were estimated. OS was analyzed in a similar fashion to PFS. The proportion of patients who respond (completely or partially) to the combination regimen was estimated and exact binomial confidence intervals were computed for the estimate. One-sample log-rank test was used to calculate the *p* value for comparing survival of the combination regimen against that of the similar population treated with nivolumab and ipilimumab. The toxicity associated with the treatment regimen (defined as at least possibly attributed to treatment) was summarized. For each type of toxicity, a patient’s worst treatment-related toxic episode was used to summarize distribution of toxicity grade experienced.

## Results

### Patients

**Figure 1:**
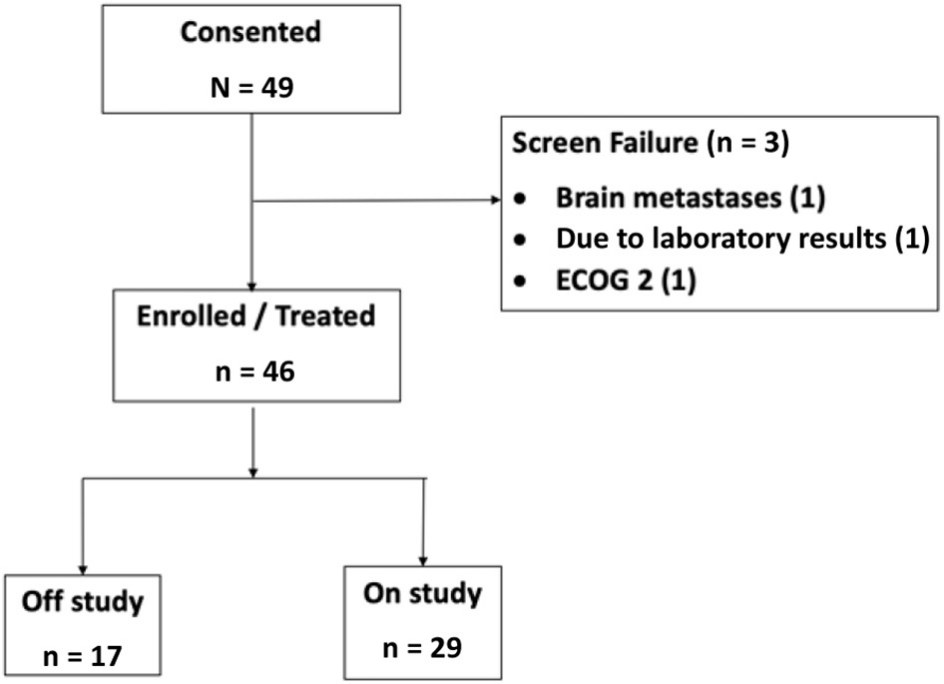
Study consort diagram. ECOG, Eastern Cooperative Oncology Group.

During the study enrolment period (October 2018–February 2021), 49 patients were consented to the trial, and ultimately 46 patients received treatment (Fig. 1). The median age was 66 (range: 48–82) years, 63% were male, and most had past or current smoking history (70% and 20%, respectively). Adenocarcinoma and squamous cell histologies were found in most tumors, 63% and 26%, respectively. There were 14 (30%) patients who had a prior history of brain metastases. KRAS mutation was the most often detected driver alteration in 17% of the patients. PD-L1 expression less than 1% was found in 21 patients (46%) and greater than or equal to 1% in 23 patients (50%). PD-L1 was not tested in 2 of 46 patients.

### Treatment

The median follow-up time from the remaining 29 alive patients was 19.0 (range: 4.8–33.4) months. The mean duration of treatment was 8.3 months with median duration of 4.4 months. As of October 2021, six patients (13%) remain on active treatment. Three patients (7%) completed the 24 months of therapy. Patients were taken off of the study related to adverse events (41%), disease progression (35%), or study withdrawal (4%).

### Outcomes

**Figure 2:**
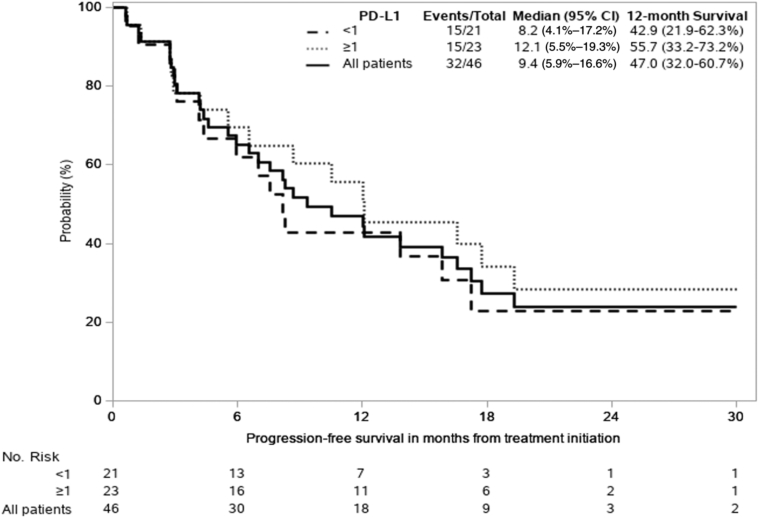
K-M Progression-free survival curve including all patients treated on study and subgroups of PD-L1 greater than or equal to 1% and less than 1%. CI, confidence interval; K-M, Kaplan-Meier; PD-L1, programmed death-ligand 1.

**Figure 3:**
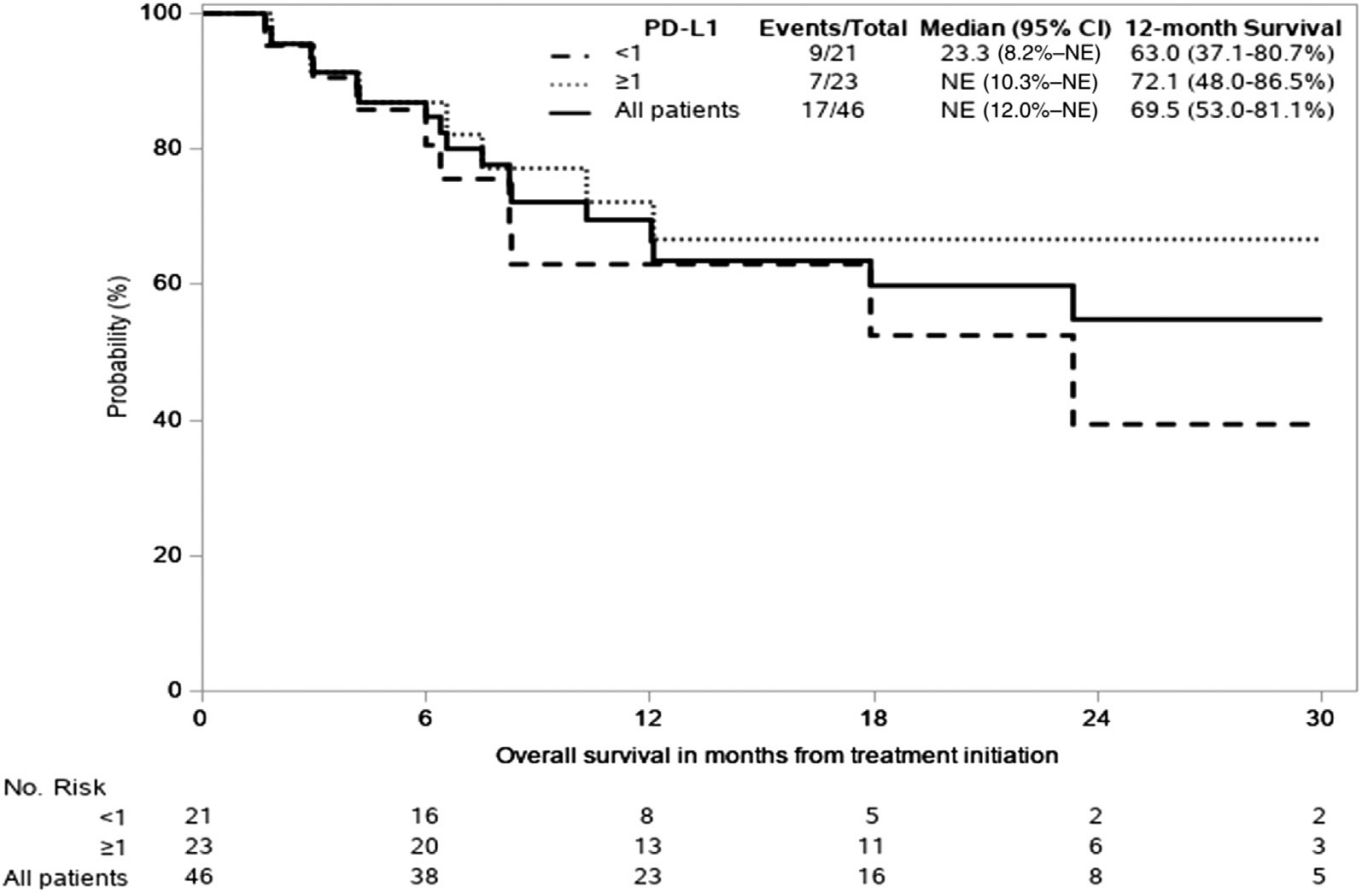
K-M of overall survival including all patients treated on study and subgroups of PD-L1 greater than or equal to 1% and less than 1%. CI, confidence interval; K-M, Kaplan-Meier; NE, not evaluable; PD-L1, programmed death-ligand 1.

**Figure 4:**
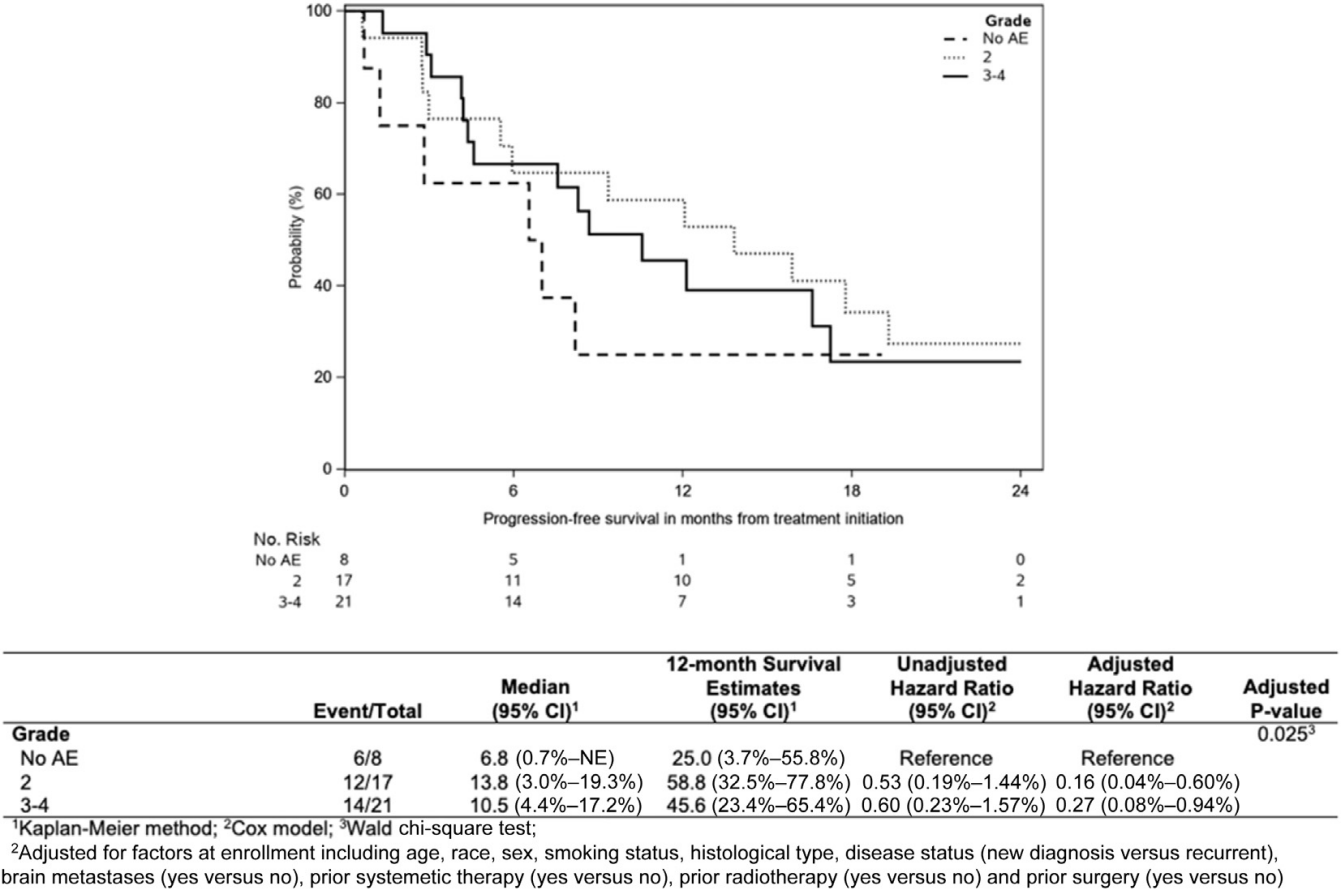
K-M curve of progression-free survival stratified by grade of immune-related adverse events. Unadjusted and adjusted Cox model hazard ratios are reported relative to reference group, in which no immune-related AEs were experienced. AE, adverse event; CI, confidence interval; K-M, Kaplan-Meier; NE, not evaluable.

The primary end point of mPFS was 9.4 months (95% confidence interval [CI]: 5.9–16.6; 80% CI: 7.6–13.8), all regardless of PD-L1 expression (Fig. 2). In patients with PD-L1 expression less than 1% (n = 21) mPFS was 8.2 months (95% CI: 4.2–17.2) and 12.1 months (95% CI: 5.5–19.3) for PD-L1 greater than or equal to 1% (n = 23). The 12-month PFS rate was 47% (95% CI: 32%–60.7%) (Fig. 2). The median OS for all patients has not yet been reached. Nevertheless, median OS in PD-L1 less than 1% patients was 23.3 months (8.2–not evaluable) and not yet mature for PD-L1 greater than or equal to 1% patients (95% CI: 10.3–not evaluable). The 12-month OS rate was 69.5% (95% CI: 53%–81%) (Fig. 3). In an exploratory analysis, no difference was found between PD-L1 expression groups (<1%, ≥1%) for either PFS or OS (unadjusted hazard ratio = 0.76 [95% CI: 0.37–1.56] and hazard ratio = 0.69 [95% CI: 0.24–1.93], respectively; adjusted for baseline variables listed in Table 1, PFS = 1.09 [95% CI: 0.40–2.93] and OS = 0.99 [95% CI: 0.91–1.07]; Table 2). In an exploratory analysis, PFS was evaluated relative to presence of immune-related toxicity. Patients experiencing grade 2 immune-related adverse event (irAE) or grade 3 to 5 had lower risk of progression, compared with patients without irAE (Fig. 4). Patients with grade 2 had lower risk of disease progression than patients experiencing grade 3 to 4, but this was not significant statistically (mPFS was 6.8, 13.8, and 10.5 mo, respectively, for patients with no irAE, versus grade 2 versus grades 3–4).

**Table 1:** Baseline Patient Demographics and Clinical Characteristics at Enrollment

Demographics	All Patients (N = 46)
Age	
Mean (SD)	66 (8)
Median (IQR)	66 (61–71)
Range	48, 82
Sex, n (%)	
Female	17 (37)
Male	29 (63)
Race, n (%)	
White	35 (76)
Black	11 (24)
Smoking status, n (%)	
Current	9 (20)
Former	32 (70)
Never	5 (11)
Histology, n (%)	
Adenocarcinoma	29 (63)
Squamous cell carcinoma	12 (26)
Adenosquamous	3 (7)
Large cell with neuroendocrine features	1 (2)
Mucinous adenocarcinoma	1 (2)
Disease status at enrollment, n (%)	
Newly diagnosed stage IV	35 (76)
Recurrence	11 (24)
Brain metastases? (yes), n (%)	14 (30)
Prior surgery? (yes), n (%)	37 (80)
Prior systemic therapy? (yes), n (%)	5 (11)
Prior radiation therapy? (yes), n (%)	23 (50)

IQR, interquartile range.

**Table 2:** Objective Response Rate and Duration of Response on Study Treatment as Found in All Patients and by PD-L1 Status

Demographics	PD-L1 < 1% (n = 21)^a^	PD-L1 ≥ 1% (n = 23)^a^	All Patients (N = 46)^a^	*p* Value
Overall response, n (%)				0.416^b^
Complete response	0 (0.0)	2 (8.7)	2 (4)	
Partial response	9 (42.9)	11 (47.8)	20 (43)	
Stable disease	10 (47.6)	7 (30.4)	19 (41)	
Progressive disease	2 (9.5)	3 (13.0)	5 (11)	
Response rate, % (95% CI)	42.9 (21.7–64.0)	56.5 (36.3–76.8)	47.8 (33.4–62.3)	0.367^b^
Duration of response (mo)				0.423^c^
n	9	13	22	
Median (IQR)	12.9 (5.7–17.8)	15.2 (9.7–18.6)	14.6 (9.1–18.6)	
Range	1.4–31.3	1.3–29.0	1.3–31.3	

Note: p value is reported from chi-square and Wilcoxon ranked sum test between PD-L1 less than 1% and greater than or equal to 1% subgroups, respectively.

CI, confidence interval; IQR, interquartile range; PD-L1, programmed death-ligand 1.

a Two patients were not tested for PD-L1.

b Chi-square test.

c Wilcoxon ranked sum test.

The ORR for patients in the study was 47.8% (95% CI: 33.4–62.3) with complete response found in two patients (4%) and partial response achieved in 20 patients (43%) (Table 1). Stable disease and tumor progression was observed in 19 (41%) and 5 (11%), respectively. The ORR was by PD-L1 status at 42.9 (95% CI: 21.7–64.0) in PD-L1 less than 1% and 56.5% (95% CI: 36.3–76.8) in PD-L1 greater than or equal to 1% expressing tumors. There was no statistical difference in ORR between PD-L1 expressing and nonexpressing groups (*p* = 0.367). The median duration of response for all patients was 14.6 months (interquartile range: 9.1–18.6).

### Safety

Treatment-related grade greater than or equal to 3 AEs was found in 54.3% of patients in the study (23 patients [50%] grade 3, one patient [2.2%] grade 4, and one patient [2.2%] grade 5). The most common treatment-related AEs are reported in Table 3. The most common reported treatment-related grade greater than or equal to 3 AEs included colitis seven (15%), pneumonitis six (13%), adrenal insufficiency five (11%), rash four (9%), infusion reaction three (7%), nephritis two (4%), and liver test elevation one (2%). Grade 2 toxicity was observed in 20 patients (43.5%) and included grade 2 hypophysitis in four patients (9%) and hypothyroidism in seven patients (15%). Furthermore, 40 patients (87%) required dose modification or treatment adjustment owing to adverse event.

**Table 3:** Most Common Treatment-Related Adverse Events Including Grades 2 to 4

Adverse Events	Grade 2 n (%)	Grades 3–4^a^ n (%)	All Patients n (%)
Fatigue	23 (50.0)	1 (2.2)	24 (52.2)
Alopecia	17 (37.0)	0 (0.0)	17 (37.0)
Anorexia	15 (32.6)	1 (2.2)	16 (34.8)
Infusion-related reaction	12 (26.1)	3 (6.5)	15 (32.6)
Adrenal insufficiency	5 (10.9)	5 (10.9)	10 (21.7)
Diarrhea	10 (21.7)	0 ( 0.0)	10 (21.7)
Rash maculopapular	5 (10.9)	4 (8.7)	9 (19.6)
Pneumonitis	2 (4.3)	6 (13.0)	8 (17.4)
Colitis	0 (0.0)	7 (15.2)	7 (15.2)
Hypothyroidism	7 (15.2)	0 (0.0)	7 (15.2)

a One grade 5 pneumothorax occurrence.

## Discussion

The results of the OPTIMAL trial reveal that the study met the primary end point by achieving mPFS of 9.4 months in treatment-naive NSCLC by using a novel regimen of nivolumab, ipilimumab, with weekly paclitaxel when compared with historical control of 5.1 months for nivolumab plus ipilimumab in CheckMate-227. To the best of our knowledge, this is the first study reporting the use of a low-dose weekly, single-agent chemotherapy added to dual immunotherapy in NSCLC. Our study has several important distinctions compared with the other frontline immunotherapy-based regimens. First, both the CheckMate-9LA and recently published Poseidon trials used a platinum doublet chemotherapy approach combined with dual immunotherapy, whereas we used a single-agent chemotherapy to improve tolerability compared with full-dose, platinum-based chemotherapy. Second, given the risk of early, on-treatment disease progression within the first several months for immunotherapy alone approaches, chemotherapy in our study was extended to four to six cycles to mitigate progression during this time. In addition, the dual immunotherapy approach maximizes the chance of prolonged duration of response as found in other studies with nivolumab/ipilimumab.^10^^,^^12^ Finally, by using a nonplatinum-based frontline regimen, patients enrolled on this study had the opportunity to receive an additional line of therapy using platinum doublet in the second-line setting.

Although the median OS has not yet been reached, the clinical outcomes from our study generally compare favorably to published reports of frontline regimens using dual anti–PD-1 or PD-L1 and anti–CTLA-4 inhibition for NSCLC. For example, in the CheckMate 227 study, the ORR was 33.1% (35.9% in PD-L1 ≥ 1% and 27.3% in PD-L1 < 1%) and mPFS of 5.1 months in both the PD-L1 expressing and nonexpressing subgroups.^9^ The 1-year survival rate in the nivolumab/ipilimumab arms for PD-L1 greater than or equal to 1% and PD-L1 less than 1% was similar between subgroups, at 63% and 60%, respectively. Two clinical trials have now also reported clinical outcomes for combination doublet chemotherapy with dual immune checkpoint blockade. In the CheckMate-9LA study, the addition of platinum-doublet chemotherapy for two cycles increased the ORR to 38.2% from 29.2% with chemotherapy alone, while increasing mPFS and OS to 6.8 months and 15.8 months, respectively.^11^^,^^13^ More recently, the Poseidon study evaluated combination durvalumab and tremelimumab with platinum-doublet chemotherapy for four cycles with option of maintenance pemetrexed for nonsquamous histologies. The mPFS and OS reported for patients treated on the Poseidon study were 6.2 months and 14.0 months, with ORR of 38.8%.^14^ In our study, the ORR and mPFS were numerically greater in the PD-L1 greater than or equal to 1% subgroup, though there was not a statistically significant difference in outcomes based on PD-L1 expression.

The toxicity profile of nivolumab and ipilimumab has been extensively described across numerous studies with large patient numbers.^15^ In the CheckMate-227 trial, any-grade diarrhea was found in 17% and greater than or equal to 3 grade was uncommon in just 1.7% of patients. Endocrinopathy of any grade was reported in 23% of patients, whereas treatment-related pulmonary toxicity was observed in 8.3% of patients.^9^ The addition of two cycles of chemotherapy to nivolumab/ipilimumab in the CheckMate-9LA study did not change the irAE profile substantively, with grade greater than or equal to 3 immune-related toxicity in 22% of patients.^11^ In the Poseidon trial, grade greater than or equal to 3 immune-related toxicity was found in 33% of patients receiving durvalumab/tremelimumab with doublet.^14^ Although the overall rate of grade greater than or equal to 3 toxicity found with combination nivolumab/ipilimumab and paclitaxel is in line with toxicity rates reported with other chemotherapy and immunotherapy studies,^4^, ^5^, ^6^ there were seemingly higher rates of irAEs in our study. Nevertheless, when interpreting the toxicity profile from the OPTIMAL study, there are multiple important considerations. First, the elevated rate of pneumonitis found in our study may likely be driven by the weekly taxane dosing rather than the immune checkpoint therapy. Weekly dosed taxane treatment has been strongly associated with increased rates of pneumonitis, specifically as high as 27% in patients with lung cancer.^16^ Second, higher rates of endrocrinopathy including thyroid dysfunction, adrenal insufficiency, and hypophysitis are generally expected with combination immunotherapy. A meta-analysis of studies including more than 7500 patients reported rates of adrenal insufficiency and hypophysitis at 4.2% and 6.4% with combination dual immune checkpoint inhibition.^17^ Furthermore, there is ample published evidence that development of endocrinopathy, including thyroid dysfunction and hypophysitis, on immune checkpoint-based treatment is predictive of improved survival for patients.^18^^,^^19^ Most recently, a pooled analysis of combination chemotherapy with atezolizumab revealed that lower grade (1–2) toxicity in general was associated with OS benefit.^20^ Ultimately, given the limited sample size in our study, it remains challenging to draw conclusions regarding specific toxicity rates from cross-trial comparisons. Nevertheless, our study indeed reveals improved PFS for patients experiencing irAE as compared with not developing toxicity. Last, infusion reactions were common in our patient cohort with most being related to paclitaxel and low grade in severity.

There are multiple described mechanisms by which paclitaxel exerts antitumor, immunostimulatory effects and could potentiate the efficacy of dual immune checkpoint blockade, as found in our study. Administration of paclitaxel, for example, has been found to increase dendritic cell maturation, interleukin-12 production, costimulatory function, and enhance antigen-specific CD-8+–mediated tumor lysis.^21^ In addition, paclitaxel facilitates immunogenic tumor cell death by promoting MHC-1 and Fas up-regulation and increasing sensitization to effector T cells.^22^ Finally, paclitaxel promotes macrophage differentiation to an antitumor and proinflammatory M1 phenotype through TLR4 signaling. Gene expression analysis before and after paclitaxel administration from patients with ovarian cancer revealed up-regulation of genes linked to effector T cell function, natural killer T cells, and macrophage activation.^23^ Preclinical data remain limited regarding the effects of paclitaxel in the setting of immune checkpoint inhibition. Nevertheless, there is substantial evidence supporting the role of other chemotherapeutics provoking immunogenic cell death, antitumor immunostimulation, and promoting response to immune checkpoint therapy.^8^ For example, carboplatin in the setting of anti–PD-L1 inhibition is known to increase effector T cells, while reducing regulatory T cells and myeloid-derived suppressor cells.^24^ The impact of dual immune checkpoint inhibition and paclitaxel on immune cell function and subpopulations will be the focus of correlative analyses of biospecimens collected at baseline and on treatment from patients treated in the OPTIMAL study.

We recognize that our study has a few important limitations. First, although statistically powered to evaluate the primary end point compared with historical control, as a single-arm phase 2 study, there was no control arm for direct comparison. As a result, comparison to larger, randomized phase 3 studies should be done with caution. In addition, the study was conducted at a single institution and may affect ability to generalize findings more broadly. Nevertheless, patients were enrolled and treated at an academic center and community campus within the institution during the course of the study. Finally, the median age (66 y) of patients is younger than the typical age demographic for advanced lung cancer and may reflect a healthier population. The other demographic characteristics are generally as expected with advanced NSCLC.

In conclusion, the combination of nivolumab and ipilimumab with paclitaxel in patients with treatment-naive, advanced NSCLC was successful in achieving the primary end point of the study by significantly improving mPFS compared with historical outcomes from nivolumab/ipilimumab alone. The regimen demonstrated a robust response rate and median OS has not yet been reached. The overall grade greater than or equal to 3 toxicity rate is similar to other chemoimmunotherapy combinations and was generally manageable. Further data are needed to understand how relative rates of irAEs compare with other immunotherapy combinations. The outcome of the OPTIMAL study provides proof-of-concept that single-agent chemotherapy can delay early disease progression, potentiate antitumor immunostimulatory effects, while sparing platinum-based chemotherapy. Other single-agent chemotherapies may be associated with less pneumonitis and potentially more favorable side effect profile, for example pemetrexed. Extensive correlative analyses are planned to interrogate the mechanisms by which paclitaxel promotes immunostimulation and potential biomarkers of toxicity and clinical benefit.

## CRediT Authorship Contribution Statement

**Jeffrey M. Clarke, Neal E. Ready:** Conceptualization, Funding acquisition.

**Jeffrey M. Clarke, Neal E. Ready, Lin Gu, Xiaofei F. Wang:** Methodology.

**Lin Gu, Xiaofei F. Wang**: Formal analysis.

**Jeffrey M. Clarke**: Writing—original draft.

**Jeffrey M. Clarke, Lin Gu, Xiaofei F. Wang, Thomas E. Stinchcombe, Marvaretta M. Stevenson, Sundhar Ramalingam, Afreen Shariff, Jennifer Garst, Andrew B. Nixon, Scott J. Antonia, Jeffrey Crawford, Neal E. Ready:** Investigation, Writing—review and editing.
